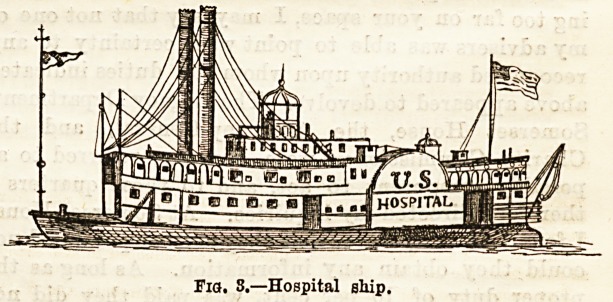# The United States Army Medical Department

**Published:** 1896-02-15

**Authors:** 


### PRACTICAL DEPARTMENTS.

### THE UNITED STATES ARMY MEDICAL DEPARTMENT.

At the Chicago Exhibition much trouble was taken by the authorities of the Medical Department of the United States Army to show by means of elaborate models what were the resources of the department at command in the war between the Northern and Southern States, and the improvements since introduced following the experience gained during that and the Franco-German struggle of the early seventies.

Complete models of hospital equipments, those used in the late war and those since evolved, were shown—canvas field hospitals, regulation post hospitals, and all the resources of surgery and first aid as called into service on the field of battle. The illustrations herewith given are from photographs taken of these models, and may be interesting to readers of The Hospital, more especially because attention has been lately directed to such matters in our preparations for the due care of sick and wounded in the Ashantee expedition, and in the recent French war in Madagascar, where, unfortunately, a breakdown in the medical service seems to have had disastrous results and added terribly to the mortality.

Specimens were also exhibited of the barrack hospitals erected during the war in America, and the transport cars in use to connect the advancing armies with the various centres supplied with full hospital accommodation. The two first diagrams illustrate an ordinary passenger railway car, fitted up for the reception and conveyance of the sick and wounded by rail, such as were attached to the army of the Cumberland. The seats were removed, and supports arranged for the reception of mattresses, each car having space for eleven beds thus, while slung over them were ordinary field stretchers, with shortened handles, providing eleven more beds. Each car could so carry twenty-two patients, and the beds in the lower tier were sufficiently wide to hold two patients in each if necessary. A very wide door was made on one side of the car to allow ample room for the ingress and egress of litters with the most severely wounded patients.

For the comfortable conveyance of the wounded in freight cars in the Franco-Prussian war, a plan was devised by a German master machinist, Grund, by name, which it has been recommended by an authority should be kept on hand in the quartermaster's department of the United States Army in any future war. This consisted in supporting field stretchers

Fig. 1.—Longitudinal section of hospital car used in the American War.

by means of transverse wooden bars resting on semi-elliptical plate springs, fixed by a spike at one end to the flooring, while at the other are rollers to permit the yielding of the spring, the latter being surmounted by U-shaped clips to receive cross-bars for slinging the litters. It is proposed that trains going to the front with provisions or ammunition

Fig. 2.— Transverse section of hospital car.

should carry a sufficient number of these springs to enable the train on its return to carry back comfortably on these improvised beds some of the sick and wounded. The arrangement would naturally a good deal diminish shaking and jarring. These springs, it has also been suggested, might profitably be used for conveying the wounded in ordinary army wagons.

Fig. 3.—Hospital ship.

Each hospital train in the American war consisted of ten or twelve passenger coaches, with several freight and baggage cars. One car was fitted up exclusively as a kitchen and store-room, and another as a dispensary, with accommodation for the medical officer in charge of the train, the steward, and an ample supply of medicine, stores, and surgical instruments and appliances of all sorts. The funnels of the engines attached to the hospital trains were painted red, and at night time distinguished by three red lanterns suspended in a row to secure their safe transport. They were never fired upon or molested in any way.

The hospital steamer "D. A. January" (depicted in sketch No. 3) was built in 1856, and was used for the transportation of the sick on the Mississippi and Ohio rivers from April, 1862, to February, 1864. She was a side-wheel steamboat of 450 tons burthen, 235 feet in length, and 65 in extreme width. Bought by the Government of the United States on the outbreak of the war in 1862, some alterations were effected, and she was at once used for the conveyance of hospital stores, in the autumn being fitted up as a general hospital with 400 beds. Assistant-Surgeon Hoff, who was in charge of the boat for a considerable time, has stated that "the Commanding General, by order, arranged the running of all hospital steamers so that they could not be interfered with by the subordinate commanders; and, once under weigh with their load of sick and wounded, were not disturbed until their destination was reached. Our flag was considered a flag of truce, fully protected us, and gave us an opportunity of keeping the hospitals always in order. . . . To overcome the difficulty as to supplies and the prompt payment of men employed on the boat, the surgeon in charge was made acting assistant-quartermaster and commissary of subsistence, this arrangement working most satisfactorily, and enabling the boat to be always in readiness to leave at a moment's notice." Every arrangement was made to have a plentiful supply of ice, a system of pipes being constructed whereby the drinking water was carried through the ice-room, and iced water conveyed to all parts of the ship. A fan ran through the entire length of the main ward, worked by machinery from below; this created a pleasant and desirable current of air, and was, besides, very efficacious in keeping away flies and mosquitoes. The "D. A. January" saw plenty of hard service during those months of war. She carried altogether 23,738 patients, of whom 530 died *en route*.

*(To be continued.)*

## Figures and Tables

**Fig. 1. f1:**
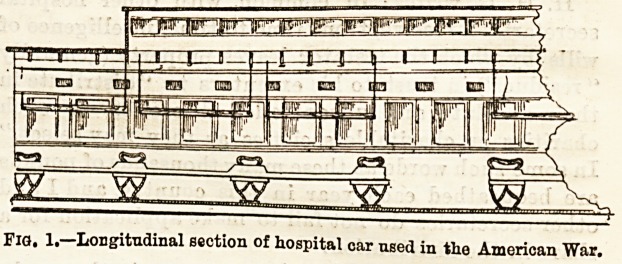


**Fig. 2. f2:**
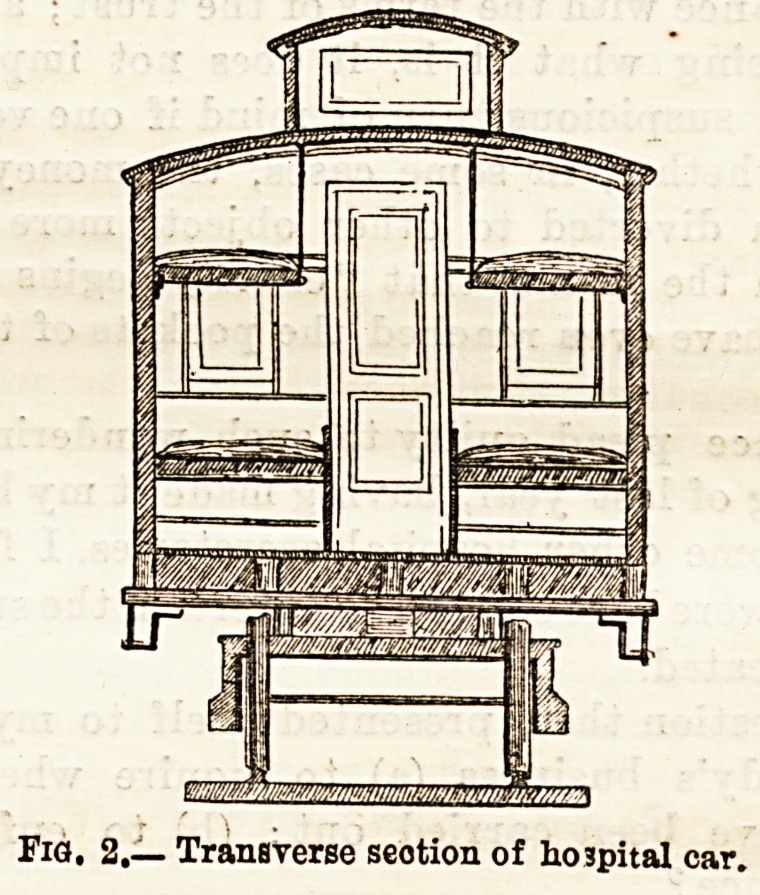


**Fig. 3. f3:**